# Dialkyl dicyanofumarates and dicyanomaleates as versatile building blocks for synthetic organic chemistry and mechanistic studies

**DOI:** 10.3762/bjoc.13.221

**Published:** 2017-10-24

**Authors:** Grzegorz Mlostoń, Heinz Heimgartner

**Affiliations:** 1 Department of Organic and Applied Chemistry, University of Łódź, Tamka 12, PL 91-403 Łódż, Poland; 2 Department of Chemistry, University of Zurich, Winterthurerstrasse 190, CH-8057, Switzerland

**Keywords:** cycloaddition reactions, electron-deficient ethenes, heterocyclization reactions, Michael additions, SET mechanism

## Abstract

The scope of applications of dialkyl dicyanofumarates and maleates as highly functionalized electron-deficient dipolarophiles, dienophiles and Michael acceptors is summarized. The importance for the studies on reaction mechanisms of cycloadditions is demonstrated. Multistep reactions with 1,2-diamines and β-aminoalcohols leading to diverse five- and six-membered heterocycles are discussed. Applications of dialkyl dicyanofumarates as oxidizing agents in the syntheses of disulfides and diselenides are described. The reactions with metallocenes leading to charge-transfer complexes with magnetic properties are also presented.

## Review

### Introduction

**Figure 1:**
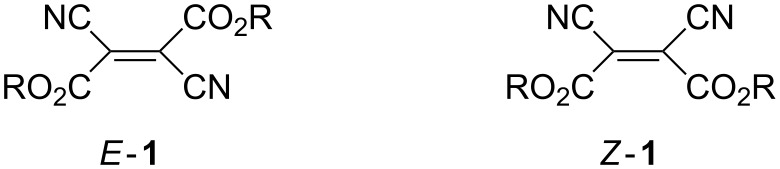
Dialkyl dicyanofumarates *E*-**1** and dicyanomaleates *Z*-**1**.

Electron-deficient alkenes form an important class of organic compounds, which are of key importance in organic synthesis. The best known representative of this class is tetracyanoethylene (TCNE) with numerous applications [1–3], and its role in cycloaddition reactions is of special interest. Dialkyl dicyanofumarates *E-***1** and dicyanomaleates *Z*-**1** ((*E*)- and (*Z*)-butenedioates **1**, Figure 1) are less known, but the availability of both stereoisomers is of great advantage for the exploration in mechanistic studies of their reactions. Similar to TCNE, these compounds are also attractive Michael acceptors with numerous applications for multistep reactions leading to heterocyclic products.

The chemistry of *E*-**1** and *Z*-**1** has not been reviewed, and the goal of the present survey is to summarize the methods of their preparation and their applications in organic syntheses. In addition, mechanistic studies of cycloaddition reactions performed with *E*- and *Z*-**1** will be discussed.

### Synthesis of dialkyl dicyanofumarates and maleates

**Scheme 1:**
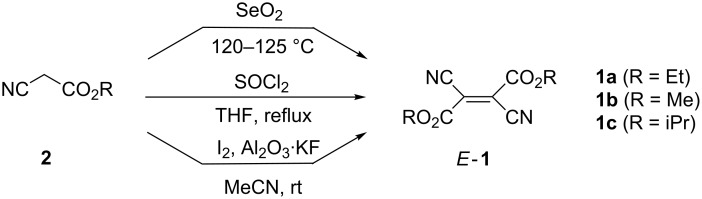
Methods for the synthesis of dialkyl dicyanofumarates *E*-**1** from alkyl cyanoacetates **2**.

Numerous methods for the synthesis of *E*-**1** have been reported and cyanoacetates as well as their bromo and dibromo derivatives are the most important starting materials. The first described method comprised the treatment of ethyl cyanoacetate (**2a**) with SeO_2_ at 120–125 °C, leading to *E*-**1a** in ca. 10% yield [4] (Scheme 1). More efficient syntheses of diverse dialkyl esters *E*-**1** were achieved in reactions of alkyl cyanoacetates **2** with SOCl_2_ in tetrahydrofuran (THF) at reflux temperature [5–8]. Unexpectedly, the attempted preparation of the di(*tert*-butyl) derivative was unsuccessful. The highest yield (90%) was obtained for *E*-**1a** when **2a** was oxidized with I_2_ in the presence of Al_2_O_3_·KF in acetonitrile at room temperature (rt) [9].

**Scheme 2:**
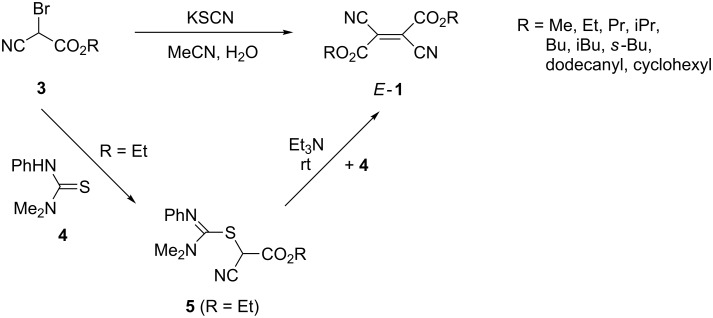
Methods for the synthesis of dialkyl dicyanofumarates *E*-**1** from alkyl bromoacetates **3**.

Another efficient method for the preparation of dialkyl esters *E*-**1** relies on the usage of alkyl bromo(cyano)acetates **3**, which upon treatment with potassium thiocyanate in aqueous acetonitrile at room temperature are converted into the corresponding *E*-**1** [10] (Scheme 2).

Alternatively, the same transformation can be performed with **3** and thiourea derivative **4** in a two-step reaction through intermediate **5** [11] (Scheme 2). The latter, upon treatment with Et_3_N, generates a carbene [:C(CN)CO_2_Et], which dimerizes to give *E*-**1a** albeit in low yield.

The dimerization of the same carbene leading to a mixture of *E*- and *Z*-**1a** was observed when ethyl dibromocyanoacetate was treated with equimolar amounts of LiI in DMF at room temperature with the highest reported yield of 83% [12]. The dimethyl ester *E*-**1b** was also obtained selectively by treatment of dimethyl 2,3-bis(hydroxyiminomethyl)fumarate with (CF_3_CO)_2_O in dioxane in the presence of pyridine at 0–10 °C [13]. The starting material was prepared in a multistep reaction from methyl β-nitropropanoate.

An efficient method for the preparation of *E*-**1b** comprises the multistep reaction of dialkylselenonium (cyano)(methoxycarbonyl)methanide with episulfides [14] or thioamides [15]. Also, methyl cyanoacetate was reported to undergo both chemical (using (NH_4_)_2_Ce(NO_3_)_6_ = CAN in methanol) or electrochemical oxidation (Ce(NO_3_)_3_, HNO_3_ in acetonitrile) yielding selectively *E*-**1b** in 68 and 77% yield, respectively [16].

However, the most efficient method for the synthesis of dialkyl dicyanomaleates *Z*-**1** is the photochemical isomerization of the corresponding *E*-isomers. The reaction is performed in dichloromethane [17] and in the presence of benzophenone [18–19] or 1,4-dicyanobenzene [20] as photosensitizer.

The comparison of the methods for the synthesis of *E*- and *Z*-**1** shows that the most convenient protocol is the treatment of the corresponding alkyl cyanoacetate with SOCl_2_ and subsequent photoisomerization of the obtained *E*-**1** into the *Z*-isomer.

### Applications in organic synthesis

#### Reactions with carbenes; cyclopropanations

**Scheme 3:**
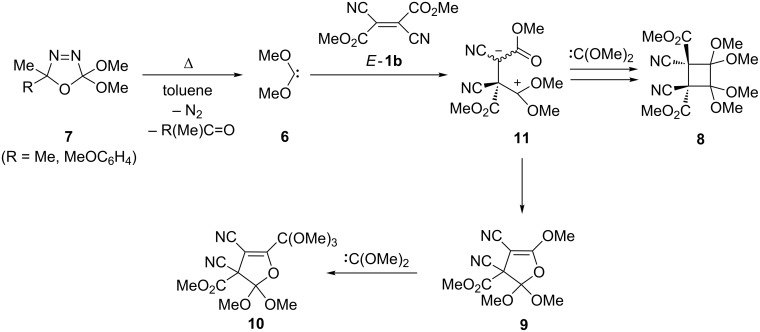
Reaction of dimethyl dicyanofumarate (*E*-**1b**) with dimethoxycarbene [(MeO)_2_C:] generated in situ from the precursor **7**.

Dimethoxycarbene (**6**), generated in situ by thermal decomposition of 2,2-dimethoxy-2,5-dihydro-1,3,4-oxadiazole **7**, reacts with *E*-**1b** yielding a mixture of the *trans*-configured tetramethoxycyclobutane **8** and 2,3-dihydrofuran **9** [21–22] (Scheme 3). The latter reacts further with dimethoxycarbene and converts into the orthoester **10** via insertion into the C–O bond. The reaction mechanism for the formation of **8** and **9** comprises the initial attack of the nucleophilic carbene onto the electron-deficient C atom of *E*-**1b** to give an intermediate zwitterion **11**. The latter undergoes two competitive reactions. The first one is the ring closure leading to **9**, and the second one involves the addition of a second carbene followed by ring closure to yield **8**.

**Scheme 4:**
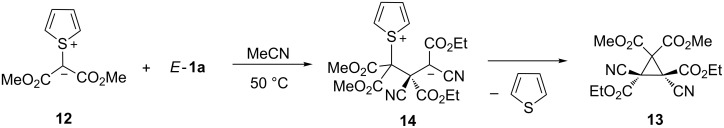
Cyclopropanation of diethyl dicyanofumarate (*E*-**1a**) through reaction with the thiophene derived sulfur ylide **12**.

The reaction with *Z*-**1b** led to the same set of products with *trans*-orientation of the substituents in **8** indicating the appearance of an intermediate zwitterion **11**. In contrast to the nucleophilic dimethoxycarbene, the formal transfer of the bis(carbomethoxy)carbene from the sulfur ylide **12** to *E*-**1a** leads to the cyclopropane derivative **13** [23] (Scheme 4). The reaction was proposed to occur stepwise via the zwiterrionic intermediate **14**.

**Scheme 5:**
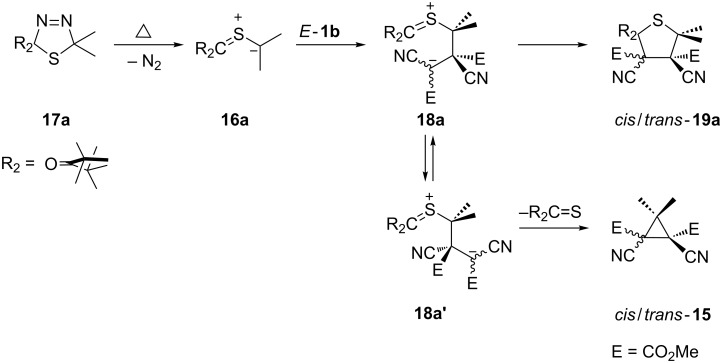
Cyclopropanation of dimethyl dicyanofumarate (*E*-**1b**) through a stepwise reaction with the in situ generated thiocarbonyl *S*-isopropanide **16a**.

Another example of a similar transfer of a carbene unit was presented to explain the formation of cyclopropanes **15** in the reaction of *E*- and *Z*-**1b** with the in situ generated thiocarbonyl *S*-isopropanide **16a**. The latter is formed through a thermal N_2_ elimination from 2,5-dihydro-1,3,4-thiadiazole **17a** [24–25] (Scheme 5). The mechanistic explanation of this cyclopropanation reaction is based on the assumption that the intermediate zwitterion **18a** undergoes either a 1,3- or 1,5-electrocyclization leading to **15** or thiolanes **19a**. The experiments with both isomers of **1b** showed that the reactions proceeded non-stereospecifically and mixtures of isomeric cyclopropanes were obtained in each case.

The *trans* and *cis*-isomers of cyclopropane **15** were also obtained in reactions of 2-diazopropane with *E*-**1b** and *Z*-**1b**, respectively [25]. In that case, the formation of the cyclopropanes was stereospecific.

The formation of mixtures of thiolanes of type **19** and cyclopropanes of type **15** in which Me_2_C is replaced by H_2_C was observed when the sterically hindered *S*-methanide derived from 2,2,6,6-tetramethylcyclohexanethione was reacted with *E*- or *Z*-**1b** [26], whereas in the cases of 2,2,5,5-tetramethylcyclopentanethione and 1,1,3,3-tetramethylindane-2-thione, respectively, only *cis*/*trans*-thiolanes were formed [27].

In contrast, the reactions of *E*- and *Z*-**1b** with di(*tert*-butyl)thiocarbonyl *S*-methanide gave ca. 1:1 mixtures of *cis/trans*-cyclopropanes as products of the CH_2_-transfer to the C=C bond in 82% yield [28]. The same products are formed in reactions with diazomethane albeit in very low yields [29].

#### [2 + 2]-Cycloadditions

**Scheme 6:**
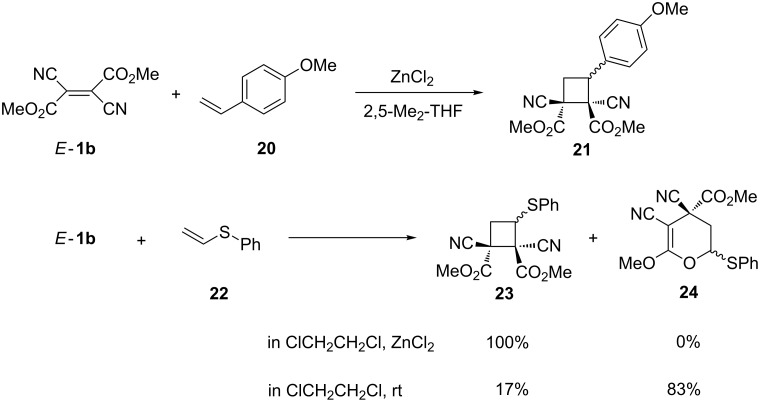
The [2 + 2]-cycloadditions of dimethyl dicyanofumarate (*E*-**1b**) with electron-rich ethylenes **20** and **22**.

The electron-deficient dicyanofumarates *E*-**1** react with electron-rich ethenes, yielding cyclobutane derivatives as product of [2 + 2]-cycloadditions. Depending on the reaction conditions and on the type of the electron-rich ethene, the reaction occurs stereoselectively or with loss of the stereochemical arrangement of substituents. For example, 4-methoxystyrene (**20**) reacts with *E*-**1b** in 2,5-dimethyltetrahydrofuran in the presence of ZnCl_2_ with complete stereoselectivity and the cyclobutane derivative **21** is formed as the sole product [30] (Scheme 6). The analogous reaction with phenyl vinyl sulfide (**22**) gave the expected cyclobutane **23** exclusively. However, in the absence of ZnCl_2_, a mixture of **23** and 3,4-dihydro-2*H*-pyran **24** was obtained, with the latter compound formed through a competitive hetero-Diels–Alder reaction [30].

The competitive formation of cyclobutane and pyran derivatives was also observed in reactions of *E*-**1b** or *Z*-**1b** with ethyl prop-1-enyl ether performed in CDCl_3_ at room temperature [31].

**Scheme 7:**
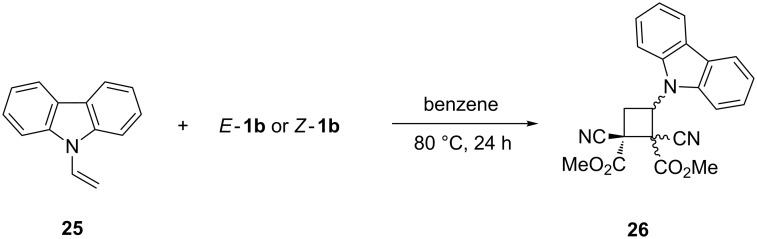
The [2 + 2]-cycloaddition of isomeric dimethyl dicyanofumarate (*E*-**1b**) and dicyanomaleate (*Z*-**1b**) with *N*-vinylcarbazole (**25**).

The reaction of *N*-vinylcarbazole (**25**) with *E*-**1b** or *Z*-**1b** in boiling benzene gave mixtures of four diastereoisomers of cyclobutanes **26** [20] (Scheme 7). The ratio of the isomers was the same irrespective of the configuration of **1b**, indicating a stepwise reaction mechanism.

**Scheme 8:**
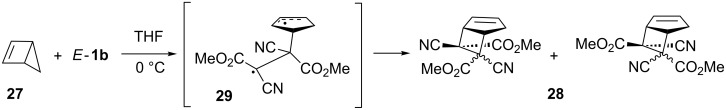
Non-concerted [2 + 2]-cycloaddition between *E*-**1b** and bicyclo[2.1.0]pentene (**27**).

Non-concerted [2 + 2]-cycloadditions were reported to occur between *E*- and *Z*-**1b** and bicyclo[2.1.0]pentene (**27**) [32]. Whereas in the case of *E*-**1b** two cycloadducts **28** were identified in the mixture, the reaction with *Z*-**1b** afforded four diastereoisomers of type **28** (Scheme 8).

The observed stereochemical outcome was explained by a diradical mechanism with isomerization of the intermediate **29** taking place only in the reaction with *Z*-**1b**. As side products isomeric homo-Diels–Alder adducts were found in the mixture.

#### [3 + 2]-Cycloadditions (1,3-dipolar cycloadditions)

**Scheme 9:**
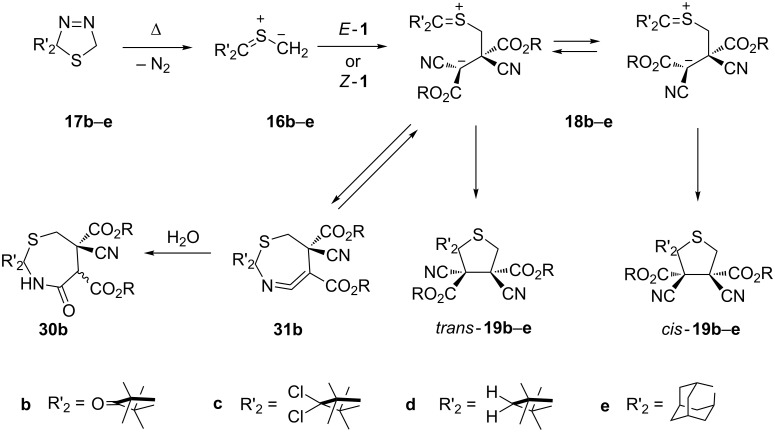
Stepwise [3 + 2]-cycloadditions of some thiocarbonyl *S*-methanides with dialkyl dicyanofumarates *E*-**1** and dicyanomaleates *Z*-**1**.

Electron-rich 1,3-dipoles such as thiocarbonyl *S*-methanides and azomethine ylides react with dipolarophiles *E*-**1** and *Z*-**1** to give five-membered cycloadducts through stepwise zwitterionic reaction mechanisms. The in situ generated sterically crowded thiocarbonyl *S*-methanide **16b** in dry THF undergoes a [3 + 2]-cycloaddition with both *E*- and *Z*-**1b** forming mixtures of diastereoisomeric thiolanes **19b** [19] (Scheme 9). However, in all studied cases, the configuration of the starting dipolarophile was preserved in the major cycloadduct. A non-concerted course of the reaction via the stabilized zwitterion **18b** was proposed to rationalize this unexpected result. In an additional experiment performed in wet THF, mixtures of diastereoisomeric, spirocyclic seven-membered lactams **30b** were isolated side by side with thiolanes **19b**. Their formation proves the appearance of the labile seven-membered ketenimine **31b**, which exists in equilibrium with zwitterion **18b**. Ketenimine **31b** is efficiently trapped with water to give **30b**.

Analogous results were obtained with different thiocarbonyl *S*-methanides **16**, generated from the corresponding precursors **17** with methyl, ethyl and isopropyl esters of type *E*- and *Z*-**1** [19,33–36]. Remarkably, the reactions of (*E*)- and (*Z*)-**1b** with thiocarbonyl *S*-methanides **16b**-**e**, in contrast to the corresponding *S*-isopropanide **16a** (Scheme 5), occurred without formation of cyclopropane derivatives.

The treatment of methyl 3-methyl-2-oxodithiobutanoate with diazomethane at −80 °C in hexane/CH_2_Cl_2_ afforded the expected 2-sulfanyl-2-isobutanoyl-2,5-dihydro-1,3,4-thiadiazole. After the addition of *E*-**1a** and warming of the mixture to room temperature, the corresponding thiolane as the [2 + 3]-cycloadduct of the intermediate thiocarbonyl *S-*methanide onto the C=C bond was obtained. The reaction was reported to occur with complete stereoselectivity [37].

**Scheme 10:**
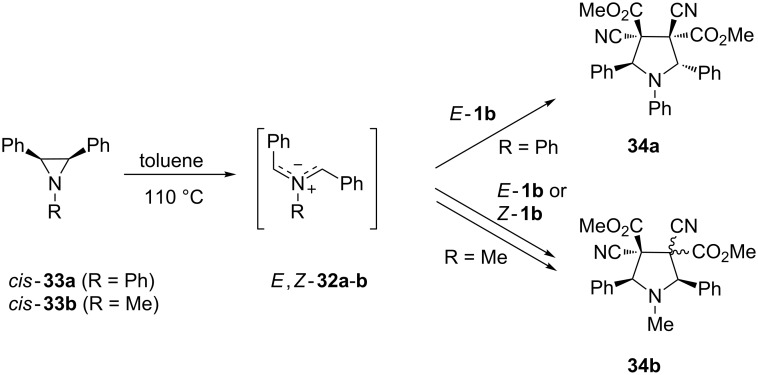
Stepwise [3 + 2]-cycloadditions of dimethyl dicyanofumarate (*E*-**1b**) and dimethyl dicyanomaleate (*Z*-**1b**) with the in situ generated azomethine ylides **32**.

Thermally generated azomethine ylides **32**, from 1,2,3-trisubstituted aziridines **33**, were tested in [3 + 2]-cycloadditions with *E*- and *Z*-**1b**, and depending on the substitution pattern of the aziridine ring, the formation of the pyrrolidine derivative **34** occurred either with complete stereoselectivity or mixtures of isomeric products were obtained. The [3 + 2]-cycloaddition of the azomethine ylide *E*,Z-**32a**, formed via conrotatory ring opening of aziridine *cis*-**33a**, with *E*-**1b** yielded cycloadduct **34a** exclusively through a concerted mechanism [38] (Scheme 10). In contrast, a more complex reaction of *cis*-1-methyl-2,3-diphenylaziridine (*cis*-**33b**) with both *E*- and *Z*-**1b** led to a mixture of stereoisomeric cycloadducts **34b**. The same mixture of products was obtained starting from *trans*-**33b**. The formation of these isomeric products suggests that in the course of the reaction, isomerizations of both the intermediate azomethine ylide of type **32** as well as of the electron-deficient dipolarophiles *E*- and *Z*-**1b**, occur. The observed experimental results were rationalized by a computational study performed at the DFT B3LYP/6-31G(d) level of theory with the PCM solvation model [39].

The thermal [3 + 2]-cycloadditions of azomethine ylides derived from diethyl *cis*- and *trans*-1-(4-methoxyphenyl)aziridine-2,3-dicarboxylates with *E*-**1b** led to isomeric pyrrolidines with preserved configuration in the fragment of the former dipolarophile [39]. Furthermore, the configuration of the ester groups at C2 and C5 corresponded with the structure of the intermediate azomethine ylide predicted for thermal ring opening of the starting aziridine. Also in this series the experimental results were confirmed by computational methods.

**Scheme 11:**
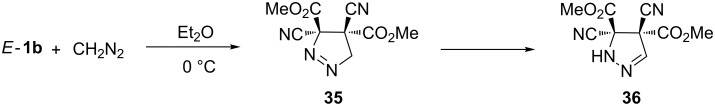
[3 + 2]-Cycloaddition of diazomethane with dimethyl dicyanofumarate (*E*-**1b**) leading to 1*H*-pyrazole derivative **36**.

In the reaction of diazomethane with *E*-**1b** in THF at room temperature 4,5-dihydro-3*H*-pyrazole **35** was detected as the initial [3 + 2]-cycloadduct by ^1^H NMR spectroscopy [29] (Scheme 11). Fast tautomerization led to the corresponding 1*H*-pyrazole **36**. The cycloaddition occurred with preservation of the configuration of the dipolarophile. In the presence of excess diazomethane the five-membered cycloadducts **35** and **36** were converted into a complex mixture of products.

In a very recent study, we demonstrated that the reactions of both *E*-**1b** and *Z*-**1b** with 9-diazofluorene at room temperature lead to mixtures of two isomeric cyclopropanes in each case. The major product obtained in the reaction with *E*-**1b** was identified as the corresponding *trans*-1,2-dicyanocyclopropane-1,2-dicarboxylate, the structure of which was established by an X-ray single crystal analysis. On the other hand, the major product isolated after the reaction with *Z*-**1b** was identified as *cis*-1,2-dicyanocyclopropane-1,2-dicarboxylate based on spectroscopic data [40]. This result suggests that the reactions follow a non-concerted pathway.

The presented studies on [3 + 2]-cycloadditions with dialkyl dicyanofumarates *E*-**1** and maleates *Z*-**1** are important from the point of view of the interpretation of reaction mechanisms of cycloadditions. The results obtained with electron-rich thiocarbonyl *S*-methanides **16** demonstrate that the classical concerted mechanism changes to non-concerted stepwise processes, which can involve zwitterionic or diradical intermediates.

#### [4 + 2]-Cycloadditions (Diels–Alder reactions)

In analogy to reactions with tetracyanoethene (TCNE), the first [4 + 2]-cycloadditions (Diels–Alder reactions) of *E*-**1a** were performed using typical 1,3-dienes such a buta-1,3-diene, isoprene, cyclopentadiene and anthracene. In all cases the reaction occurred at elevated temperature and afforded the expected cycloadducts in good to excellent yields [41]. The problem of the configuration of the substituents has not been discussed, but it seems likely that the homogeneous products are *trans*-configured. This assumption is supported by the results obtained with cyclopentadiene and both *E*- and *Z*-**1b** [32]. Whereas the reaction with *E*-**1b** led to only one stereoisomer, in the case of *Z*-**1b**, *exo*- and *endo-*isomers with preserved *cis-*orientation of the CN and CO_2_Me substituents were formed.

**Scheme 12:**
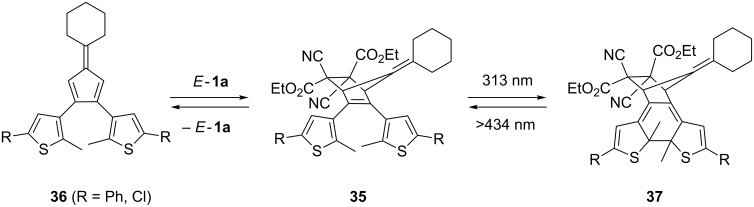
Reversible Diels–Alder reaction of fulvenes **36** with diethyl dicyanofumarate (*E*-**1a**).

Polycyclic products **35** were prepared through a concerted [4 + 2]-cycloaddition of *E*-**1a** with thiophene-functionalized fulvenes **36** [42] (Scheme 12). These cycloadducts are reported to undergo reversible intramolecular photocyclization to give products of type **37**.

**Scheme 13:**
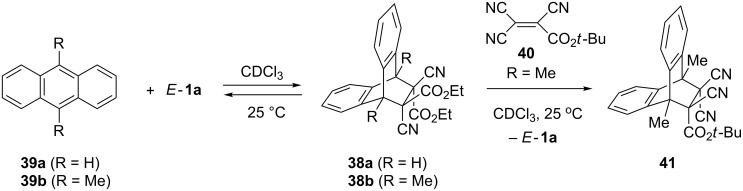
[4 + 2]-Cycloaddition of 9,10-dimethylanthracene (**39b**) and *E*-**1a**.

The formation of the thermolabile [4 + 2]-cycloadducts **38** was observed in reactions of some dialkyl dicyanofumarates *E*-**1** with anthracene (**39a**) and 9,10-dimethylanthracene (**39b**) [8,43] (Scheme 13). In an experiment with **38b** and *tert*-butyl tricyanoacrylate (**40**) in CDCl_3_ at 25 °C an exchange of the dipolarophile leading to **41** was observed by ^1^H NMR spectroscopy [8]. Similar systems were prepared on solid phase and used as a new molecular recognition system [44].

**Scheme 14:**
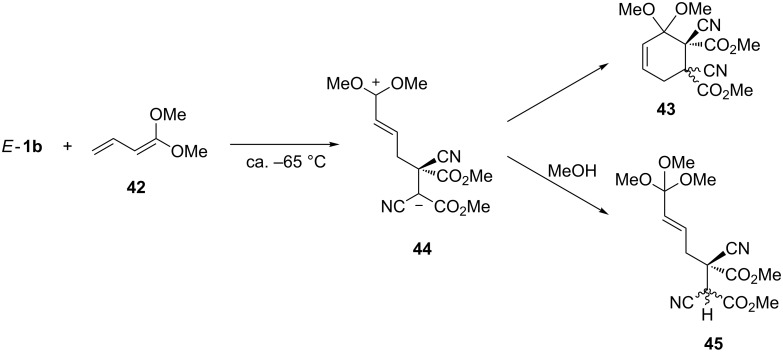
Stepwise [4 + 2]-cycloaddition of dimethyl dicyanofumarate (*E*-**1b**) with electron-rich 1,1-dimethoxy-1,3-butadiene (**42**) through the intermediate zwitterion **44**.

The [4 + 2]-cycloaddition reactions of *E*- and *Z*-**1b** with electron-rich 1,3-dienes have been studied extensively by Sustmann and collaborators. Thus, 1-methoxybuta-1,3-diene reacted with both dienophiles in a stereospecific manner, and in both cases mixtures of two stereoisomeric cyclohexenes with preserved stereochemistry in the dienophile fragment were obtained [45]. On the other hand, reactions with 1,1-dimethoxybuta-1,3-diene (**42**) led, in both cases, to similar mixtures of cycloadducts **43**, however, with loss of stereochemistry of the used dienophiles (Scheme 14). These results were explained by a stepwise reaction mechanism proceeding through zwitterion **44** as an intermediate. This hypothesis was confirmed by a trapping experiment with MeOH, which afforded 1,1,1-trimethoxy derivative **45**.

In analogy to 1-methoxybuta-1,3-diene, 1-(dimethylamino)buta-1,3-diene was reacted with *E*- and *Z*-**1b** in CH_2_Cl_2_ at −50 °C yielding a single cycloadduct, which exists in equilibrium of two conformers as characterized by ^1^H NMR spectroscopy [46]. In the case of *Z*-**1b**, the initial isomerization to *E*-**1b**, induced by the basic nature of the Me_2_N group, is a likely explanation for the observed result.

In extension of the study with amino-substituted electron-rich 1,3-dienes, reactions were also performed with 1,4-bis(dimethylamino)buta-1,3-diene. These required low temperature (ca. −50 °C) to avoid the formation of complex product mixtures. Based on the ^1^H NMR analysis, only one product, identical in reactions with *E*- and with *Z*-**1b**, was formed [47]. Also bis(dimethylamino)-substituted 1,3-dienes including bicyclic representatives were used for reactions with *E*- and *Z*-**1b** [48–49]. Herewith the formation of the [4 + 2]-cycloadducts occurred non-stereospecifically and mixtures of stereoisomeric products resulting from a stepwise mechanism were obtained. In order to find proofs for the postulated reaction pathways, supporting kinetic, spectroscopic and computational studies were carried out [45,48,50–51].

A stepwise reaction has also been suggested for the formal [4 + 2]-cycloaddition of *E*- and *Z*-**1b** with ethyl prop-1-enyl ether leading to a mixture of the corresponding 3,4-dihydro-2*H*-pyran and cyclobutane derivatives in a non-stereospecific manner [31].

**Scheme 15:**
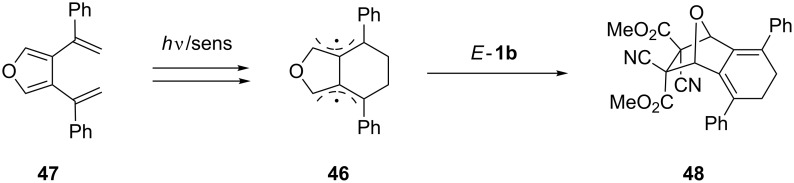
Formal [4 + 2]-cycloaddition of 3,4-di(α-styryl)furan (**47**) with dimethyl dicyanofumarate (*E*-**1b**).

A mechanism with the radical intermediate **46** governs the formal [4 + 2]-cycloaddition reaction of *E*-**1b** with 3,4-di(α-styryl)furan (**47**, Scheme 15). The photoinduced reaction occurs via an electron-transfer (PET) process and led to the formation of the polycyclic product **48** in a stereospecific manner [52]. Similar products were obtained as well with less electron-deficient dienophiles such as dimethyl fumarate and maleic anhydride.

#### Michael-type reactions

**Scheme 16:**
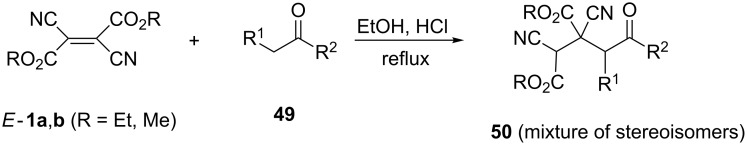
Acid-catalyzed Michael addition of enolizable ketones of type **49** to *E*-**1**.

Enolizable ketones **49** react with *E*-**1** in ethanolic solution in the presence of HCl to yield, after heating for 5 h, the corresponding Michael adduct **50** in 68–76% yield [53] (Scheme 16). These reactions were performed using both acyclic and cyclic ketones. In the case of dimedone, the reaction was carried out in ethanol at room temperature overnight [54].

Remarkably, *N,N*-dialkylanilines in DMF solution at 50–60 °C also react with *E*-**1a** as Michael donors to give diethyl 2-(4-dialkylaminophenyl)-2,3-dicyanosuccinates [55]. Heating these adducts with aqueous Na_2_CO_3_ solution results in elimination of HCN leading to the corresponding ethene derivatives.

**Scheme 17:**
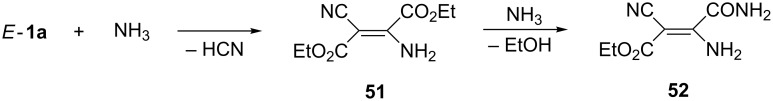
Reaction of diethyl dicyanofumarate (*E*-**1a**) with ammonia NH_3_.

The electron-deficient dialkyl dicyanofumarates *E*-**1** undergo smooth reactions with *N*-nucleophiles, such as ammonia, primary and secondary amines, hydrazine, and carbohydrazides. In some of these reactions, the initially formed adducts, after subsequent elimination of HCN, undergo heterocyclization (see chapter Heterocyclization reactions). The reaction of *E*-**1a** with either aqueous ammonia or gaseous NH_3_ in acetonitrile leads to the enamines **51** [56] (Scheme 17). Further reaction with excess NH_3_ gives rise to the corresponding monoamide **52**. Analogous reactions with differently substituted anilines and β*-*naphthylamine, respectively, afforded the corresponding enamines of type **51**, when the amine was used in excess.

On the other hand, the reactions of *E*-**1a** and an aromatic amine in a ratio of 2:1 led, unexpectedly, to the formation of another enamine with two cyano and one ester group. Notably, in the case of 4-nitroaniline, no reaction was observed. However, in another publication, the formation of an enamine of type **51** was described when *E*-**1a** was treated with the 4-nitroaniline anion in DMSO [57].

**Scheme 18:**
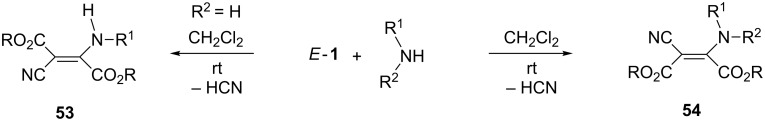
Reaction of dialkyl dicyanofumarates *E*-**1** with primary and secondary amines.

Diverse primary and secondary amines were reacted with dialkyl dicyanofumarates *E*-**1** in CH_2_Cl_2_ at room temperature (molar ratio ca. 1:1), and enamines containing two ester and one cyano group were formed as exclusive products in good to high yields. The X-ray analysis showed that products **53** obtained with primary amines are *Z*-configured whereas those derived from secondary amines, **54**, display *E*-configuration [58] (Scheme 18). The observed different configurations demonstrate the importance of the intramolecular hydrogen-bond between the NH and ester groups.

**Scheme 19:**
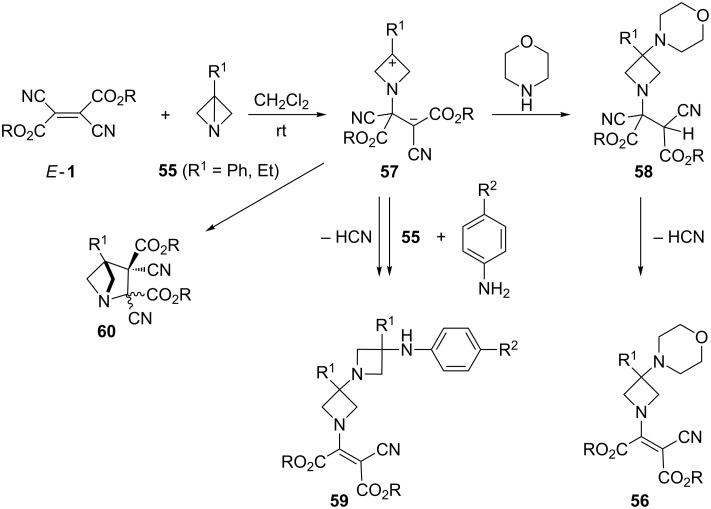
Reaction of dialkyl dicyanofumarates *E*-**1** with 1-azabicyclo[1.1.0]butanes **55**.

The reactions of *E*-**1** with morpholine performed in the presence of equimolar amounts of strained 1-azabicyclo[1.1.0]butanes **55** afforded enamines **56** containing both amine units [58] (Scheme 19). Their structures evidence that the first reaction step is the Michael-type reaction of **55** leading to a zwitterionic intermediate **57**, which is trapped by morpholine to give the adduct **58**. The latter eliminates HCN and converts to enamine **56**. The postulated pathway was supported by a similar experiment, in which methanol replaced morpholine as a trapping reagent [58–59]. An interesting observation was made when less nucleophilic anilines were used as trapping agents. The formation of adducts of type **56** proceeds in competition with the trapping reaction by a second molecule of **55** leading to the homologue enamine **59** [60].

Finally, reactions performed with **55** in the absence of any trapping reagent yielded 1-azabicyclo[2.1.1]hexanes **60** as products of an intramolecular cyclization of the intermediate zwitterion **57**. In all cases, these products were obtained as mixtures of *cis*-and *trans*-stereoisomers in favor of the *trans*-isomer [59,61].

Another class of nucleophilic reagents used for reactions with *E*-**1b** is hydrazine and its derivatives. The parent hydrazine used as the hydrate reacts with *E*-**1** in ethanol at room temperature, and in the case of diisopropyl dicyanofumarate (*E*-**1c**), the crystalline enehydrazine was isolated in 82% yield [62]. In addition, the *N*-benzyl-protected (*S*)-proline hydrazide took part in the reaction in CH_2_Cl_2_ solution at room temperature, and after the addition–elimination sequence, the corresponding enehydrazide, analogous to enamines **53/54**, was obtained in 56% yield [63]. The configuration of this enehydrazide has not been proved, but the course of its heterocyclization suggests the *E*-configuration (see following chapter).

#### Heterocyclization reactions

Due to the presence of six electrophilic centers, dialkyl dicyanofumarates *E*-**1** are useful starting materials for reactions with dinucleophilic reagents, which in one-pot procedures lead to diverse heterocyclic products (tandem reactions). These reactions occur through an initial formation of the Michael-type adduct followed by a heterocyclization step upon involvement of either a cyano or an ester group as a second electrophilic center.

**Scheme 20:**
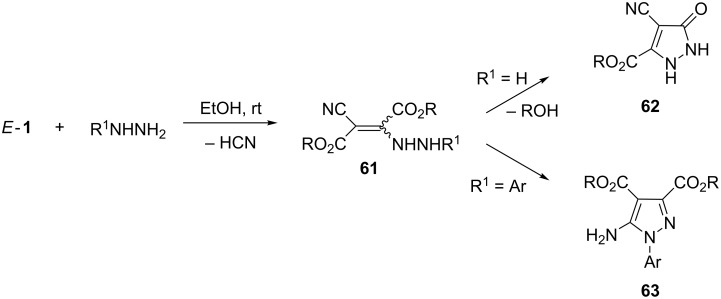
Formation of pyrazole derivatives in the reaction of hydrazines with *E*-**1**.

Hydrazine is a powerful dinucleophile and reacts easily with *E*-**1** in ethanol at room temperature yielding, after spontaneous elimination of HCN, the corresponding enehydrazine derivatives **61** [62] (Scheme 20). In case of the sterically less crowded *E*-**1a**,**b** these products immediately undergo a heterocylization through a selective attack of the NH–NH_2_ group onto the vicinal *Z*-oriented ester group. The only products obtained are 1,2-dihydropyrazole-3-carboxylates **62** in good yield.

Interestingly, reactions with arylhydrazines afforded, in the presence of ammonium or sodium acetate, 5-aminopyrazole-3,4-dicarboxylates **63** [64] (Scheme 20). Apparently, in these cases, the intermediate enehydrazines **61** undergo an alternative heterocyclization involving the cyano group. This result suggests that the *E*-configuration should be attributed to these enehydrazines **61** (R^1^ = Ar).

**Scheme 21:**
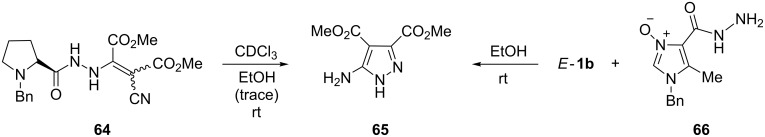
Formation of 5-aminopyrazole-3,4-dicarboxylate **65** via heterocyclization reactions.

Carbohydrazides were widely applied for reactions with *E*-**1**. The enehydrazide **64**, derived from *N*-benzylproline hydrazide, in the presence of ethanol, undergoes a cyclization reaction yielding the aminopyrazole **65** in 44% yield [63] (Scheme 21). The explanation of the reaction course is based on the assumption that under the reaction conditions ethanolysis of the carbohydrazide occurs and the formed enehydrazine **61** (R^1^ = H) converts into product **65**. The analogous reaction sequence was observed when *E*-**1b** was treated with hydrazide **66** in ethanolic solution at room temperature. These results show that enehydrazides of type **64**, very likely, exist as *E*-isomers, because the enehydrazine formed after ethanolysis has to be *E*-configured to enable cyclization with the CN group.

An analogous reaction pathway was also described for the formation of 5-amino-1-carbamoylpyrazole-3,4-dicarboxylates from dialkyl dicyanofumarates *E*-**1** and semicarbazide hydrochloride in boiling ethanol solution in the presence of sodium acetate [64]. The yields of the products are in the range of 57–72%.

**Scheme 22:**
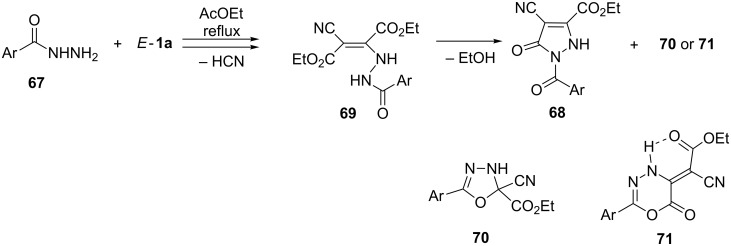
Reactions of aryl- and hetarylcarbohydrazides **67** with *E*-**1a**.

A different type of heterocyclization was reported in the reactions of carbohydrazides **67** derived from benzoic acid and some hetarylcarboxylic acids with *E*-**1a** [65–66]. In these cases, the formation of mixtures of two products is described, and in both cases pyrazol-3-ones **68** were obtained in ca. 35% yield (Scheme 22). The heterocyclization leading to **68** occurs in the intermediate enehydrazide **69** through the nucleophilic attack of the N-atom onto the ester group. The second product was postulated either as a 2,3-dihydro-1,3,4-oxadiazole-2-carboxylate **70** [65] or 2-(6-oxo-4*H*-1,3,4-oxadiazin-5(6*H*)-ylidene)acetate **71** [66].

The reaction of *E*-**1a** with 1-benzyl-1-phenyl or 1-allyl-2,5-dithiobiurea in THF at 20 °C is reported to produce, via multistep conversions, mixtures of 6*H*-1,3,4-thiadiazine derivatives containing only half of the starting fumarate [67].

**Scheme 23:**
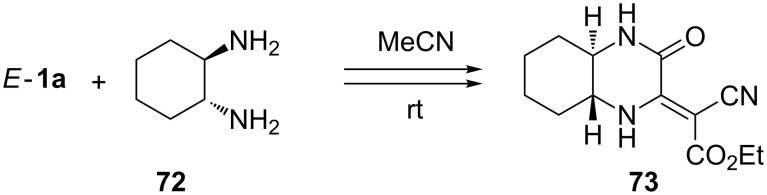
Multistep reaction leading to perhydroquinoxaline derivative **73**.

Differently substituted piperazin-2-ones can be efficiently prepared by reacting dialkyl dicyanofumarates *E*-**1** with alkane or cycloalkane-1,2-diamines. For example, the reaction with *trans*-cyclohexane-1,2-diamine (**72**) performed in acetonitrile at room temperature for 30 min gave the bicyclic piperazine (quinoxaline) derivative **73** in 61% yield [68] (Scheme 23). The analogous reaction with [(*S*)-pyrrolidin-2-yl]methylamine ((*S*)-prolinamine) with *E*-**1a**–**c** occurred smoothly in CH_2_Cl_2_ at room temperature yielding optically active (4-oxohexahydropyrrolo[1,2-*a*]pyrazin-3-ylidene)-2-cyanoacetates as single stereoisomers (79% yield) [63].

The same reaction pathway was observed in reactions with aromatic 1,2-diamines. For example, starting with benzene-1,2-diamine, the corresponding 2-oxo-1,2,3,4-tetrahydroquinoxaline derivatives, analogous to **73**, were obtained in high yields as *Z*-isomers exclusively [69]. In the case of the isomeric 2,3- and 3,4-diaminopyridines, the initial addition reaction occurred via the attack of the more nucleophilic NH_2_ group at the 3-position [69].

**Scheme 24:**
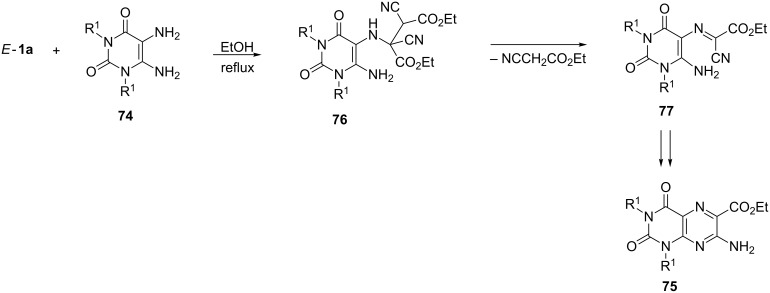
Synthesis of ethyl 7-aminopteridin-6-carboxylates **75** via a domino reaction.

Less nucleophilic 1,2-diamines, such as 5,6-diaminouracil and -thiouracil or 1,2-diaminobenzimidazole, react with *E*-**1** in a different way, and 7-aminopteridin-6-carboxylates are formed. For example, 5,6-diaminouracil (**74**, R^1^ = H) and *E*-**1a** react in boiling ethanol within 1 h to give the heterocyclic product **75** (R^1^ = H) in 65% yield [70] (Scheme 24). The mechanistic interpretation of this conversion comprises the elimination of cyanoacetate instead of HCN from the primary adduct **76**. The subsequent heterocyclization of **77** occurs via nucleophilic attack of the NH_2_ group onto the CN group, and H-migration then leads to the products **75**.

**Scheme 25:**
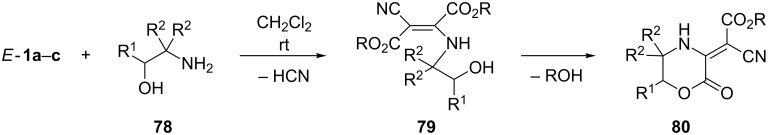
Synthesis of morhpolin-2-ones **80** from *E*-**1** and β-aminoalcohols **78** through an initial aza-Michael addition and subsequent heterocyclization step.

Another class of dinucleophilic reagents rarely used in reactions with *E*-**1** is that of β-aminoalcohols. The different nucleophilicity of the NH_2_ and OH groups determines the sequence of the reaction steps. In the reported cases, the initially formed *N-(*β*-*hydroxyalkyl)enamines **79** undergo spontaneous lactonization with the geminal alkoxycarbonyl group leading to the six-membered morpholin-2-one derivatives **80** as the only products [62] (Scheme 25). In one case, the stereochemical structure of the product was established by X-ray determination.

**Scheme 26:**
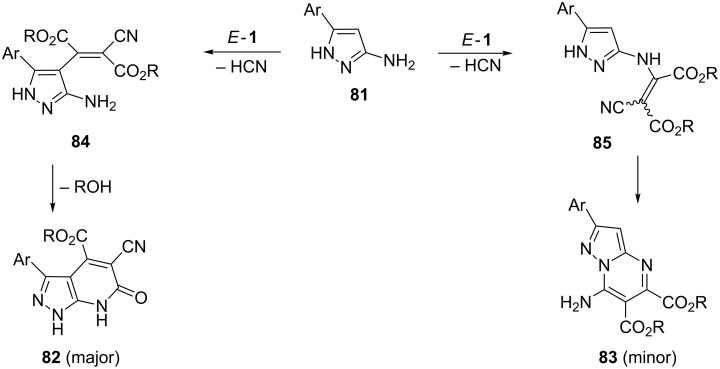
Reaction of 3-amino-5-arylpyrazoles **81** with dialkyl dicyanofumarates *E*-**1** via competitive nucleophilic attack of C4 or NH_2_.

An interesting reaction course was observed in the reaction of *E*-**1** with 3-amino-5-arylpyrazoles **81**, which are known to react as N or C nucleophiles. The type of the products obtained depended on the reaction conditions. Whereas heating in 1,2-dichloroethane (85 °C) or stirring in DMF at room temperature afforded mixtures of hetero-bicyclic products **82** and **83** in favor of **82**, reactions performed in DMF at 100 °C led to **82** as the sole product [6] (Scheme 26). The formation of the products is initiated by the nucleophilic attack of either C4 or NH_2_ of **81** onto *E*-**1**, followed, in both cases, by elimination of HCN to give intermediates **84** and **85**, respectively. The heterocyclization leading to **82** is a lactamization, whereas a ring closure via N attack onto the terminal cyano group in **85** results in the formation of the minor product **83**.

**Scheme 27:**
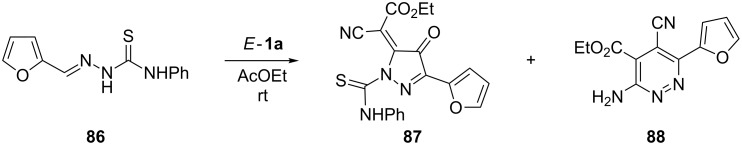
Heterocyclization reaction of thiosemicarbazone **86** with *E*-**1a**.

Another example of a heterocyclization, which occurs with participation of an N- and a C-nucleophile was reported for the reaction of *E*-**1a** and thiosemicarbazone **86**, derived from furfural. This reaction, performed in ethyl acetate at room temperature, led to a mixture of pyrazolone **87** and pyridazine **88** in favor of the former compound (54%) [71] (Scheme 27).

**Scheme 28:**
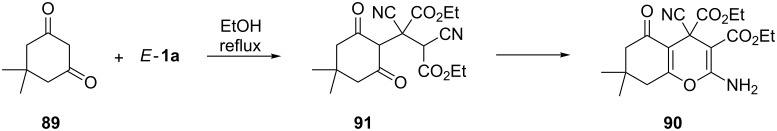
Formation of diethyl 4-cyano-5-oxotetrahydro-4*H*-chromene-3,4-dicarboxylate (**90**) from *E*-**1a** via heterocyclization reaction.

Heating equimolar amounts of *E*-**1a** and dimedone (**89**) in ethanol at reflux for 8 h afforded 4*H*-pyran **90** as the heterocyclization product of the initially formed succinate **91** (see chapter Michael type reactions) [54] (Scheme 28). In this case, the reaction pathway comprises the ring closure through the attack of the O-nucleophile onto the terminal cyano group. The analogous reaction was reported for the enolizable indane-1,3-dione.

#### Redox reactions

**Scheme 29:**
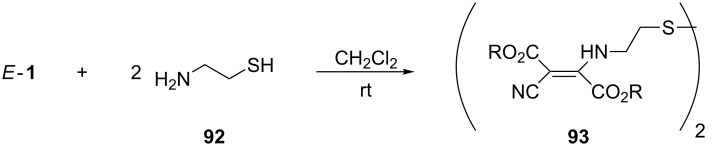
Reaction of dialkyl dicyanofumarates *E*-**1** with cysteamine (**92**).

The replacement of β*-*aminoalcohols by β*-*aminothiols in reactions with dialkyl dicyanofumarates *E*-**1** led, unexpectedly, to products containing a disulfide group together with enamine units. For example, the reaction with cysteamine (**92**) with *E*-**1a** in CH_2_Cl_2_ at room temperature gave the disulfide **93** in 42% yield [72] (Scheme 29).

**Scheme 30:**
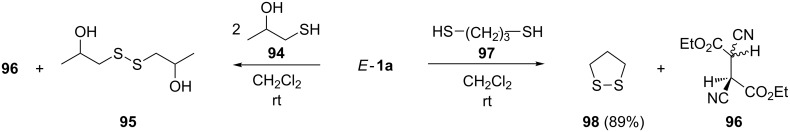
Formation of disulfides through reaction of thiols with *E*-**1a**.

Similar reactions performed with β*-*mercaptoalcohols, e.g., 1-mercaptopropan-2-ol (**94**), took place smoothly at room temperature, and the corresponding dihydroxydisulfides (e.g., **95**) were formed side-by-side with diethyl 2,3-dicyanosuccinates (**96**, Scheme 30). Finally, when 1,2-dithiols of type **97** were subjected to reactions with *E*-**1a** under the same reaction conditions, cyclic disulfides **98** were obtained as products of a redox reaction [72].

The analogous reaction course was observed with selenols, which were converted into the corresponding diselenides in good yields.

The formation of disulfides and diselenides is explained as a redox process through a single-electron transfer (SET) mechanism with *E*-**1a** as the oxidizing reagent, which converted into a mixture of diastereoisomeric diethyl dicyanosuccinates **96** [72]. Very likely, an analogous SET mechanism governs also the reaction of *E*-**1** with diethyl phosphite in boiling 1,2-dichloroethane leading to the dialkyl dicyanosuccinates **96** [7].

#### Charge-transfer (CT) complexes

**Scheme 31:**
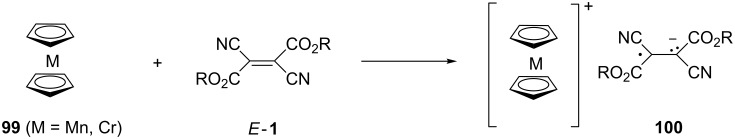
Formation of CT salts of *E*-**1** with Mn^2+^ and Cr^2+^ metallocenes through one-electron transfer.

Dialkyl dicyanofumarates *E*-**1**, similar to tetracyanoethylene (TCNE), are well-known as one-electron acceptors. They react with metallocenes **99**, such as manganocenes [73–74] and chromocenes [75], to form one-to-one charge-transfer salts **100** (Scheme 31), which are molecular magnets. Their physical properties depend on the size of the alkyl groups of the ester function [73]. Some chromocene complexes with *E*-**1a**,**b** were studied by means of X-ray crystallography [74,76].

Dialkyl dicyanofumarates *E*-**1** form also CT complexes with electron-rich non-conjugated dienes such as hexamethyl Dewar benzene [77–78], as well as indene and acenaphthylene [79]. These complexes were used for photochemical studies, e.g., the photoisomerization of Dewar benzenes into the corresponding aromatic systems.

#### Miscellaneous reactions

**Scheme 32:**
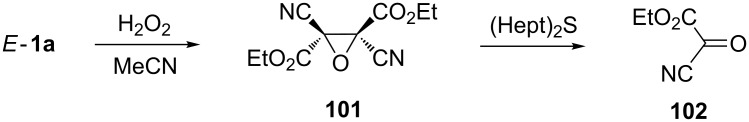
Oxidation of diethyl dicyanofumarate (*E*-**1a**) with H_2_O_2_ to give oxirane **101**.

The oxidation of *E*-**1a** with H_2_O_2_ in acetonitrile gave oxirane **101**, which subsequently was used for reactions with diverse nucleophiles [80] (Scheme 32). Upon treatment with diheptyl sulfide, **101** was transformed into ethoxalyl cyanide (**102**) [81].

**Scheme 33:**
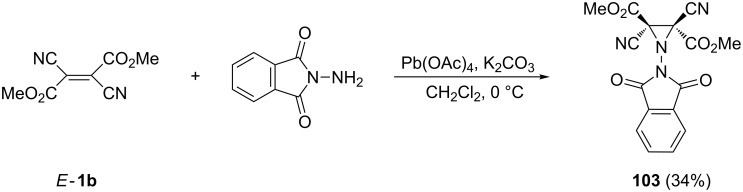
The aziridination of *E*-**1b** through nitrene addition.

The aziridination of *E*-**1b** through the addition of in situ generated aminonitrene leading to aziridine **103** bearing the phthalimido residue at the N-atom was also reported [82] (Scheme 33).

## Conclusion

Dialkyl dicyanofumarates and dicyanomaleates, which belong to the group of strongly electron-deficient alkenes, are versatile building blocks for the synthesis of diverse carbo- and heterocycles through [2 + 2], [3 + 2] and [4 + 2]-cycloaddition reactions, functionalized with ester and cyano groups. The high ability for stabilization of radical and carbanionic centers leads, in many instances, to the violation of the classical concerted cycloaddition mechanisms. The accessibility of both stereoisomers offers a unique opportunity to prove mechanistic pathways experimentally. Furthermore, dialkyl dicyanofumarates are good Michael acceptors and easily react with N- and C-nucleophiles. Their reactions with dinucleophiles, such as 1,2-diamines and β-aminoalcohols are of special importance as they offer access to a variety of heterocycles through an addition–elimination–heterocyclization sequence. Their ability to act as single-electron acceptors, demonstrated in the reaction with thiols and selenols, allows their application as oxidizing reagents. In addition, this property allows the preparation of charge-transfer salts with manganocenes and chromocenes, which are of interest as molecular magnets.
